# Actinium-225-PSMA versus lutetium-177-PSMA radioligand therapy for metastatic castration-resistant prostate cancer: results of an observational study

**DOI:** 10.3389/fonc.2026.1744007

**Published:** 2026-03-10

**Authors:** Tatiana Yu Kochetova, Mikhail B. Potievskiy, Lidia A. Nekrasova, Valeriy V. Krylov, Peter V. Shegai, Sergei A. Ivanov, Andrei D. Kaprin

**Affiliations:** 1 National Medical Research Radiological Centre of the Ministry of Health of the Russian Federation, Obninsk, Russia; 2 Department of Oncourology, Moscow City Oncological Hospital №. 62, Moscow, Russia; 3 Department of Urology and Operative Nephrology, Peoples’ Friendship University of Russia (RUDN University), Moscow, Russia

**Keywords:** actinium-225-PSMA, hematological toxicity, lutetium-177-PSMA, metastatic castration-resistant prostate cancer, overall survival, radioligand therapy

## Abstract

**Background:**

Prostate-specific membrane antigen (PSMA)-targeted radioligand therapy (RLT) with Lutetium-177 (^177^Lu) and Actinium-225 (^225^Ac) is increasingly used in metastatic castration-resistant prostate cancer (mCRPC), but head-to-head prospective data remain limited.

**Methods:**

We conducted an observational study of mCRPC patients treated with ^177^Lu-PSMA (n=116; 2022–2023) or ^225^Ac-PSMA (n=43; 2023). Primary outcomes were PSA response (≥50% decline) and overall survival (OS); secondary outcomes included hematologic toxicity. Treatments were given every 8 ± 2 weeks (1–6 cycles; median 2) with administered activity typically 5–10 GBq (median 7.5 GBq) alongside standard androgen-deprivation therapy; concurrent chemotherapy was not allowed.

**Results:**

Median follow-up was 9 months (^177^Lu-PSMA) and 10 months (^225^Ac-PSMA). Median OS was 13.0 months (95% CI 9.5–18.3) for ^177^Lu-PSMA and 11.8 months (95% CI 7.0–NR) for ^225^Ac-PSMA, with no significant difference between groups. A ≥50% PSA decline occurred in 42.2% (^177^Lu-PSMA) and 40.5% (^225^Ac-PSMA). Receiving >2 RLT courses was associated with longer OS in both cohorts (^177^Lu-PSMA: 18.3 vs 7.3 months; ^225^Ac-PSMA: OS not reached vs 5.2 months). Trends toward worse outcomes were observed in patients with visceral (especially hepatic) metastases and in those previously exposed to taxanes. Hematologic toxicity was frequent but mostly grade 1–2: anemia 66% (^177^Lu-PSMA) vs 58% (^225^Ac-PSMA), leukopenia 59% vs 57%, thrombocytopenia 47% vs 48%; treatment-related deaths were not observed.

**Conclusions:**

In this observational experience, ^225^Ac-PSMA and ^177^Lu-PSMA achieved comparable survival and PSA response with predominantly mild-to-moderate hematologic toxicity. Greater treatment exposure (>2 cycles) correlated with improved survival. Randomized trials are warranted to refine sequencing and patient selection for PSMA-RLT.

## Introduction

Radioligand therapy (RLT) using radiopharmaceuticals targeting prostate-specific membrane antigen (PSMA) is a new treatment option for patients with metastatic castration-resistant prostate cancer (mCRPC). The most studied agent for radioligand therapy (RLT) is ^177^Lu-PSMA, which demonstrated an advantage in using ^177^Lu-PSMA in combination with standard anti-tumor therapy excluding chemotherapy and PARP inhibitors in comparison to standard anti-tumor therapy as monotherapy in patients with mCRPC exhibiting high 68Ga-PSMA accumulation in metastatic lesions, who had received at least one line of chemotherapy and one line of next-generation androgen receptor-targeted therapy before inclusion in the study (1). Another potentially more effective radiopharmaceutical for treating mCRPC is ^225^Ac-PSMA (2), but data regarding its efficacy to date are restricted to phase II clinical trials and retrospective studies. Despite significant interest in RLT and its widespread application, questions remain regarding the optimal role of RLT among other treatment modalities for mCRPC. The advantages of ^225^Ac-PSMA as a first-line RLT treatment are still under debate (2).

Data from studies on the advantages of early RLT are contradictory; however, a meta-analysis of single-arm studies (mostly retrospective), indicated the absence of prior chemotherapy as a favorable prognostic factor for patients receiving ^177^Lu-PSMA therapy (3). Taxane-naïve patients were more likely to experience a greater than 50% reduction in PSA levels, with increased progression-free survival and overall survival (3). The randomized phase II trial TheraP was conducted to compare the efficacy of ^177^Lu-PSMA therapy and cabazitaxel (4). The study found that ^177^Lu-PSMA had an advantage in terms of PSA response, but no differences in overall survival were detected during subsequent follow-up. A statistically significant reduction in overall survival was observed in patients who did not undergo screening for the study due to low 68Ga-PSMA accumulation in pathological lesions (5). There was also no overall survival advantage for taxane-naïve patients treated with ^177^Lu-PSMA compared to those who underwent docetaxel therapy in a phase II randomized study (5), although a reduction in PSA levels greater than 50% was more frequently observed in patients within the ^177^Lu-PSMA arm.

Phase III randomized studies are underway regarding the use of ^177^Lu-PSMA in taxane-naïve patients. However, in the PSMAfore study (6), ^177^Lu-PSMA is compared against switching from one line of next-generation androgen receptor-targeted therapy to another; unfortunately, researchers did not aim to compare PSMA therapy directly with docetaxel chemotherapy, which is the gold standard treatment for patients with mCRPC and high tumor burden (7).

In addition to chemotherapy, RLT compete with 223Ra therapy for patients with predominant skeletal involvement and no visceral or brain metastases with high 68Ga-PSMA accumulation (8). To date, we have not found randomized studies addressing the advantages of one method over the other or the optimal sequence of therapies in this regard. The RaLu study demonstrated the safety of administering RLT with ^177^Lu-PSMA following the completion of 223Ra therapy (8). A retrospective multicenter study including 416 patients, among whom 85 received 223Ra, analyzed various types of therapy as factors potentially affecting patient survival following the initiation of ^177^Lu-PSMA therapy. In this study, as well as in the aforementioned meta-analysis, a statistically significant overall survival advantage was shown for taxane-naïve patients compared to those who received one or two lines of chemotherapy. No statistically significant difference in overall survival was detected between patients who received 223Ra before ^177^Lu-PSMA therapy and those who did not receive 223Ra.

## Materials and methods

### Study design and participants

This is an observational (real practice) study of patients with histologically diagnosed prostate adenocarcinoma conducted at the A.F. Tsyb Medical Radiological Research Center (MRRC) from 2022 to 2023. All patients included in the study were treated for progressing mCRPC. Progression was defined as an increase in serum prostate-specific antigen (PSA) levels over three consecutive measurements taken at intervals of no less than 7 days, with a PSA level greater than 5 ng/mL, or according to radiologic progression criteria – new metastatic lesions and/or an increase in the size of lesions based on RECIST 1.1 criteria (4). An essential criterion for inclusion was the presence of PET-CT imaging with either 18F-PSMA or 68Ga-PSMA; one patient was included based on SPECT-CT data with 99mTc-PSMA. There were no restrictions on age or prior treatment. Patients undergoing ^225^Ac-PSMA treatment underwent PET-CT after 2–3 courses and at the end of therapy. Patients receiving ^177^Lu-PSMA underwent SPECT-CT before each course and at the end of treatment.

The study was conducted in accordance with the principles of the Declaration of Helsinki. The research involved the analysis of anonymized clinical data, and all patients had previously provided written informed consent for the standard medical treatment from which the data were derived. Ethical Committee of FSBI “National Medical Research Radiological Centre” reviewed and approved this study (Protocol ID: 02/01-22) and confirmed that no any specific informed consent was required.

### Procedures

Radiopharmaceuticals used in the study consisted of an aqueous solution based on the chemical precursor transport molecule DOTA-PSMA, labeled with the radionuclides lutetium-177 or actinium-225, prepared for intravenous administration shortly before being given to patients. The treatment was performed with in-house isotopes in accordance with 61 Federal Law of Russian Federation. Patients received courses with an interval of 8 ± 2 weeks. The administered activity was determined by the investigator based on the overall health status of the patient and the presence of risk factors, ranging from 5 to 10 GBq, with a median of 7.5 GBq. RLT was performed alongside the best standard oncology treatment, which included continuous androgen deprivation therapy using LHRH analogs or bilateral orchiectomy; the use of next-generation antiandrogens was permitted. Concurrent chemotherapy with RLT was not allowed.

### Outcomes and assessments

Primary outcomes were PSA response (defined as a ≥50% decline from baseline) and overall survival (OS). Secondary outcomes included hematologic toxicity, graded according to CTCAE criteria. Data on hematologic parameters and PSA levels were collected after each treatment cycle. OS was defined as the time from the first cycle of RLT to death from any cause.

### Statistical analysis

Statistical analysis was performed using appropriate statistical software. Continuous variables were described as medians with interquartile ranges, and categorical variables as counts and percentages. Survival analysis was performed using the Kaplan-Meier method, and groups were compared with the log-rank test. A p-value of <0.05 was considered statistically significant. Correlation analysis was performed using Kendall’s tau.

## Results

The study analyzed the experience of the A.F. Tsyb Medical Radiological Research Centre (MRRC). It reviewed the results of therapy using ^177^Lu-PSMA RLT in 116 patients from 2022 to 2023, and ^225^Ac-PSMA RLT in 43 patients who received treatment in 2023. The characteristics of the patient groups regarding the presence of different therapeutic methods in their medical history are presented in **Table 1**.

**Table 1:** Baseline clinical parameters.

Parameter	^225^Ac-PSMA (n=42)	^177^Lu-PSMA (n=116)	p-value
Age, median	68,5 (66; 71)	69 (64; 72)	0.180
PSA start, median	244,4 (72; 526)	168,8 (46; 830)	0.834
Concomitant treatment	12 (28,6%)	56 (48,3%)	0.004
Taxane	32 (76,2%)	55 (47%)	0.002
Abiraterone	9 (21,4%)	13 (11,2%)	0.168
Enzalutamide	12 (28,7%)	18 (15,5%)	0.106
Apalutamide	0	6 (5,2%)	
Olaparib	7 (16,7%)	8 (7%)	0.123
223Ra	14 (33,4%)	21(18,1%)	0.069
Bone metastases	39 (92,8%)	109 (94%)	0.907
Lymph node metastases	32 (76,2%)	86 (74,1%)	0.956
Visceral metastases	14 (33,4%)	35 (30,2%)	0.853

**Figure 1:**
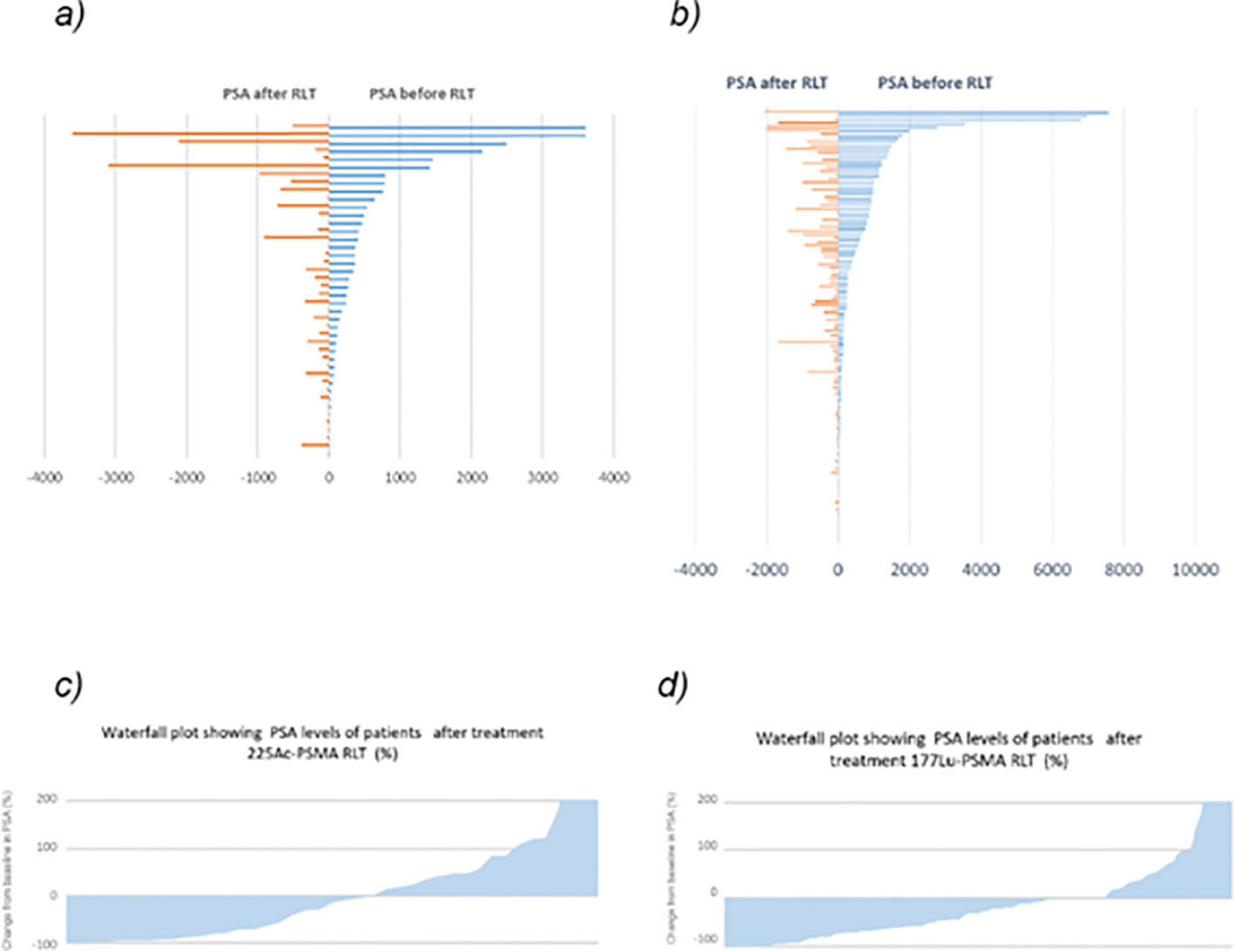
**(a)** Change in PSA levels of patients during treatment ^225^Ac-PSMA; **(b)** Change in PSA levels of patients during treatment ^177^Lu-PSMA RLT **(c)** Waterfall plot showing PSA levels of patients after treatment ^225^Ac-PSMA RLT (%) **(d)** Waterfall plot showing PSA levels of patients after treatment ^177^Lu-PSMA RLT (%).

A total of four patients (3%) had PSA levels below 5 ng/mL in the group treated with ^177^Lu-PSMA, and two patients (5%) in the group treated with ^225^Ac-PSMA RLT. The maximum PSA level in patients receiving ^177^Lu-PSMA reached 7582 ng/mL, (median 168,8 ng/mL (46; 830). In patients treated with ^225^Ac-PSMA it was 3607 ng/mL,(median - 244.4 ng/mL (72; 526). Change in PSA levels of patients during treatment ^225^Ac-PSMA and ^177^Lu-PSMA RLT is presented in **Figure 1**. All patients included in the study exhibited moderate to high PSMA accumulation according to PET-CT data in all clinically significant lesions. A separate analysis of the cohort with liver metastases revealed 8 patients in ^177^Lu-PSMA group (6,8%) and 5 (12%) in ^225^Ac-PSMA group with liver metastases. Due to the limited number of patients, this subgroup was not analyzed separately. The study also analyzed data from 43 patients who received therapy with ^225^Ac-PSMA; among them, 26 patients had a history of treatment with ^177^Lu-PSMA. Patients who were treated under the ^177^Lu-PSMA RLT treatment protocol were not included in the ^225^Ac-PSMA RLT study. The administered activity ranged from 6 to 12 GBq, with treatment conducted repeatedly at intervals of 8 ± 2 weeks, and the number of administrations varied from 1 to 6, with a median of 2 administrations for both therapeutic agents.

**Figure 2:**
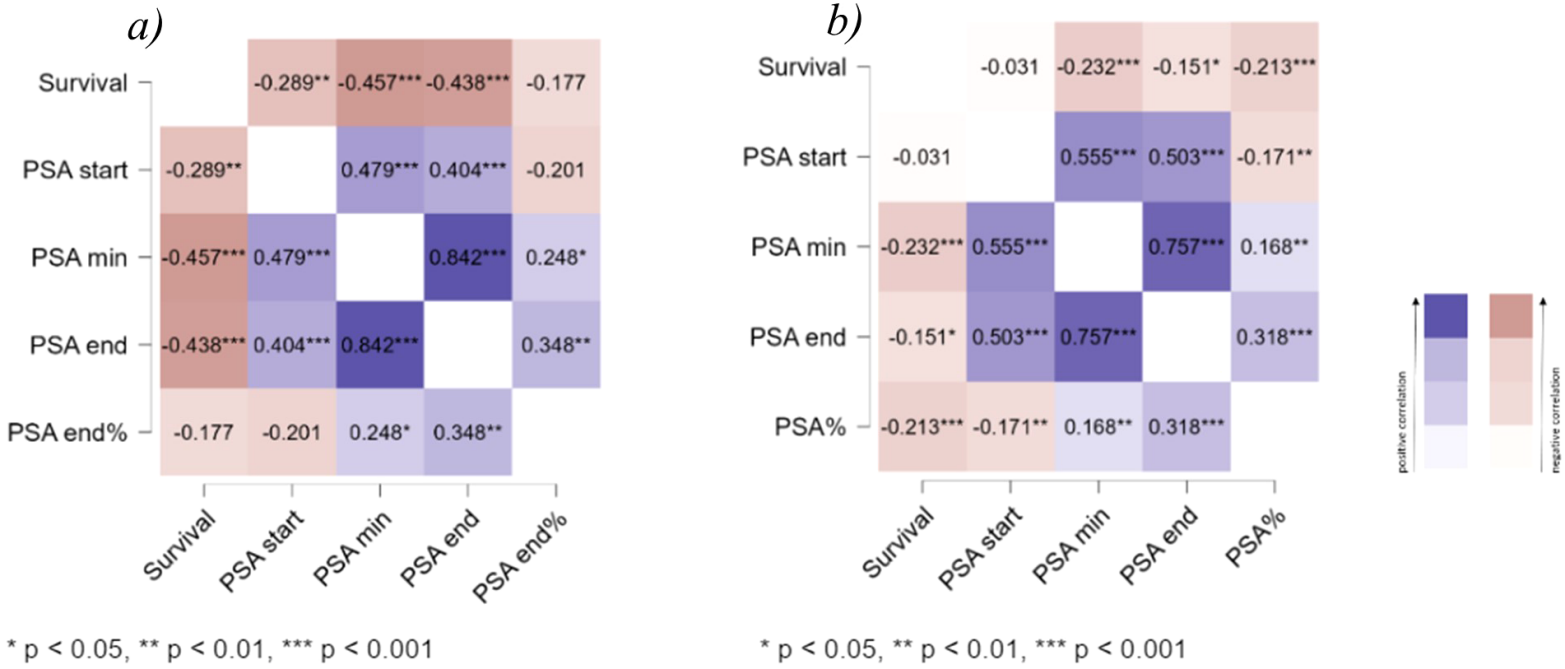
Kaplan-Meier survival plots for ^225^Ac-PSMA RLT **(a)** and ^177^Lu-PSMA RLT **(b)**.

Median follow-up was 10 months for patients receiving ^225^Ac-PSMA RLT and 9 months for those receiving ^177^Lu-PSMA RLT. Median overall survival was 11.8 months (95%Cl 7.002- nd) in the ^225^Ac-PSMA group and 13.0 months (95%Cl 9.5, 18.3) in the ^177^Lu-PSMA RLT group (**Figure 2**). All deaths were due to mCRPC and its complications. No treatment-related deaths were reported.

**Figure 3:**
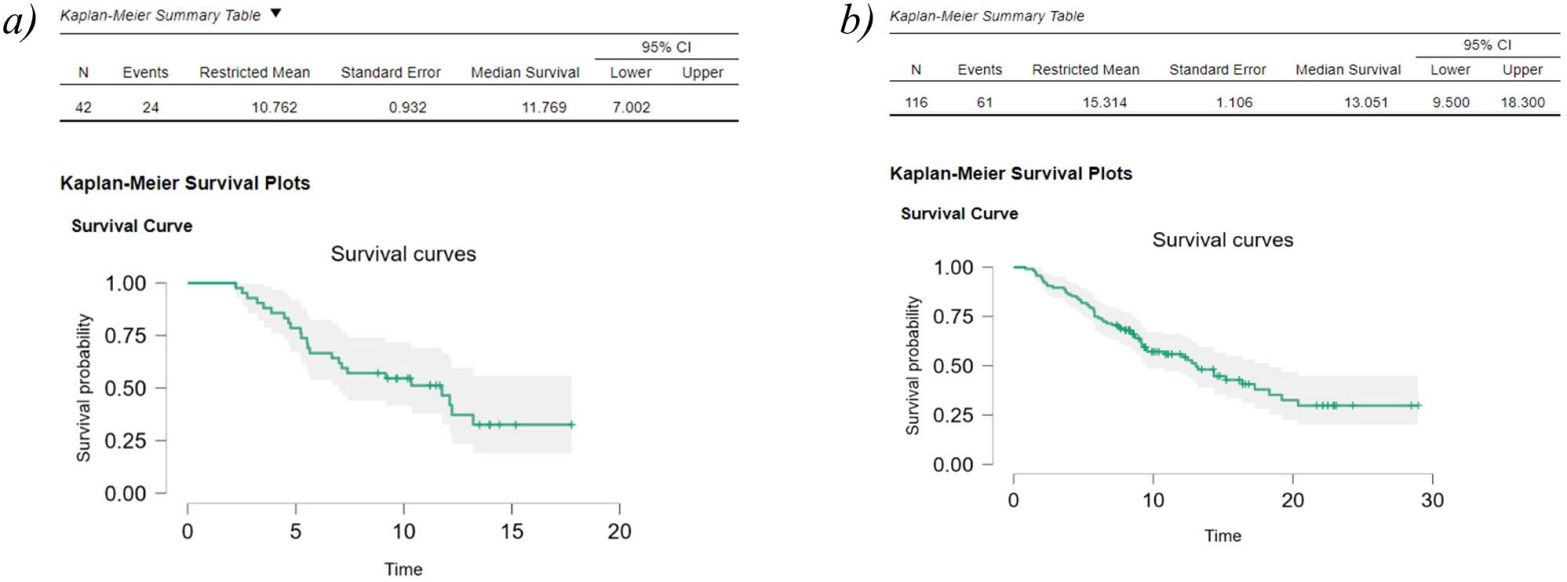
Kendall’s tau heatmap for ^225^Ac-PSMA RLT **(a)** and ^177^Lu-PSMA RLT **(b)**.

According to the correlation analysis of survival and PSA level in the ^225^Ac-PSMA group, the most significant correlation was found with PSA level at the end of treatment and to a lesser extent with PSA level at the start of treatment. A similar pattern was observed in the ^177^Lu-PSMA RLT group. These results are in good agreement with already known data and clinical experience. However, the correlation between PSA elimination and survival was not pronounced in the group ^225^Ac-PSMA RLT (**Figure 3**).

Median overall survival was significantly higher in patients who had a PSA reduction of at least 50%, a trend observed in both the ^225^Ac-PSMA RLT and ^177^Lu-PSMA RLT groups. In the ^225^Ac-PSMA RLT group, a PSA reduction of 50% or more was observed in 17(40.5%) and in the ^177^Lu-PSMA RLT group in 50 (42.2%) patients. This may also be because ^225^Ac-PSMA RLT is used for treatment after another RLT at a later stage of treatment. Median overall survival in the group was 6.7 months and 13.2 months for patients with no PSA reduction and with a 50% or greater reduction. In the ^177^Lu-PSMA RLT group, median overall survival was 6.7 and 14.3 months without and with a 50% PSA reduction.

In our study, there was an increase in median overall survival in subgroups of patients who received more than 2 courses of RLT. Thus, in the group ^177^Lu-PSMA RLT median overall survival of patients who received more than 2 courses was 18.3 months and 7.3 months in those patients who have received 1–2 courses of treatment. In the ^225^Ac-PSMA RLT group, the median survival was not reached in the longer treatment subgroup, in the 1–2 courses subgroup median OS was 5,2 months.

Also, a trend toward increased overall survival for both the ^225^Ac-PSMA and ^177^Lu-PSMA RLT groups was observed in the subgroups without liver and visceral metastases. However, this difference was not significant in the ^225^Ac-PSMA RLT group, also because of the small number of patients.

Previous 223Ra-chloride treatment was not significantly associated with an increase in median overall survival in patients in the ^177^Lu-PSMA RLT group; in the ^225^Ac-PSMA RLT group, the median survival was not reached.

Patients who received treatment (enzalutamide, abiraterone, olaparib) during radioligand therapy had decreased overall survival in both groups, but this difference was not significant.

Previous taxane chemotherapy in both the ^177^Lu-PSMA RLT and ^225^Ac-PSMA RLT groups, although a trend toward decreased OS in the previous taxane chemotherapy subgroups was observed. Previous studies have reported that the use of taxane-based chemotherapy in patients has a negative impact on overall survival; therefore, these patient subgroups require further study.

There was no significant difference in overall survival when comparing the results of ^225^Ac-PSMA RLT and ^177^Lu-PSMA RLT. In the subgroup analysis, significant differences were found only when comparing the subgroup of patients who did not experience toxicity after the first course of treatment, in which case survival was higher in the ^1777^Lu-PSMA RLT group. The results of the analysis are presented in **Table 2**.

**Table 2:** Median overall survival for patient subgroups.

Overall survival									
^225^Ac-PSMA RLT				^177^Lu-PSMA RLT				^225^Ac-PSMA RLT vs ^177^Lu-PSMA RLT	
Median, 95%Cl		log-rank test	p-value	Median, 95%Cl		log-rank test	p-value	Log-rank test	p-value
PSA decline of ≥50%									
Yes	13.215 (11.769, nd)	5.036	0.025	Yes	NR	14.549	<. 001	0.571	0.450
No	6.673 (4.767, nd)			No	9.067 (6.667, 13.051)			0.691	0.406
More than 2 courses of treatment									
Yes	NR	16.154	<. 001	Yes	18.300 (13.182, nd)	17.353	<. 001	0.182	0.670
No	5.260 (4.471, nd)			No	7.330 (5.720, 10.767			1.240	0.265
Visceral metastasis									
Yes	7.267 (5.654, nd)	1,504	0.220	Yes	9.139 (5.367, nd)	4.381	0.036	0.249	0.618
No	11.769 (7.002)			No	14.333 (10.767, nd)			0,620	0,431
Previous 223Ra									
Yes	NR	4,347	0.037	Yes	15.200 (10.767, nd)	1.610	0.204	0.015	0,901
No	7.068 (5.523, nd)			No	12.767(9.139-18.300			2.994	0.084
Olaparib									
Yes	NR			Yes	14.333 (9.167, nd)	0.066	0.797	0.240	0.624
No	11.770 (6.670, nd)			No	13.051 (9.500, 20.367)			1.096	0.295
Concomitant treatment									
Yes	8.286 (5.523, nd)	0.831	0.362	Yes	12.767 (12.097, nd)	1.067	0.302	3.514	0.061
No	12.130 (6.673, nd)			No	13.051 (8.667, 19.200)			0.048	0.826
Previous taxane									
Yes	9.172 (5.556, nd)	0.320	0.572	Yes	12.459 (9.139, 16.367)	1.285	0.257	0.401	0.527
No	12.229 (7.400, nd)			No	18.300 (9.270, nd)			0.929	0.335
Hematological toxicity after 1st course									
Yes	NR	0.004	0.952	Yes	8.448 (6.312, nd)	6.792	0.009	0.172	0.678
No	11.770 (7.000, nd)			No	17.258 (17258, nd)			7.089	0.008

In patients in the ^177^Lu-PSMA RLT group with PSA below 5 ng/mL at the start of treatment, two cases of disease progression were observed after 4 courses, one patient died after one course of treatment, one patient discontinued treatment due to toxicity, and one patient was unavailable for data collection on disease dynamics assessment. In the ^225^Ac-PSMA RLT group, both patients with PSA below 5 ng/mL progressed after 2 and 3 courses.

To obtain additional data on the impact of PSA dynamics on treatment outcomes, patients who received ^177^Lu-PSMA RLT treatment were divided into subgroups. In the subgroup of patients whose PSA decreased by 50% or more as a result of treatment (n=41) (n=23) (n=25) 46% of patients remained under observation or continued ^177^Lu-PSMA RLT without progression, 36% of patients progressed, 12% of patients discontinued treatment due to toxicity, and 2 patients (5%) died during the inter-course period. In the group with PSA changes from -50 to +25% (n=23), 61% of patients progressed, 22% discontinued treatment due to toxicity, 1 patient (5%) remained under observation, and 3 patients (13%) died. In the subgroup where PSA increased by more than 25% (n=25), 84% of patients progressed, 2 patients (8%) remained under observation, and 2 patients continued treatment due to the absence of radiological progression and stabilization of their condition despite an unsatisfactory response according to PSA data (both patients underwent 4 courses of treatment and, according to follow-up examinations and somatic status, disease stabilization was observed despite an increase in PSA of more than 50% from the baseline value).

**Figure 4:**
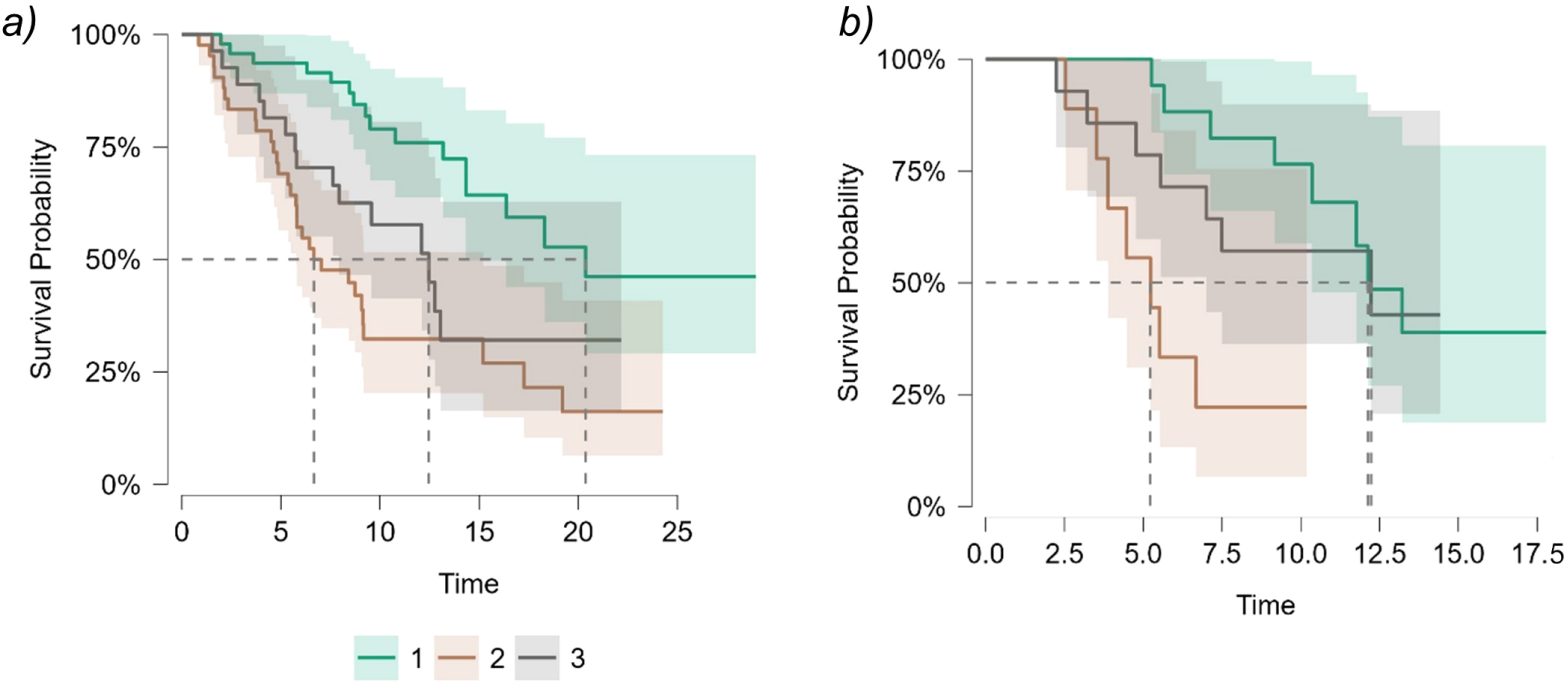
Overall survival in patients receiving in subgroups depending on PSA dynamics: ^177^Lu-PSMA RLT **(a)**; ^225^Ac-PSMA RLT **(b)**. Green - PSA decreased by 50% or more. Orange - PSA changed in the range from -40 to +25%. Gray - PSA increased by more than 25%.

Patients receiving ^225^Ac-PSMA RLT were divided into similar subgroups. In the first subgroup (n=17, PSA decreased by 50%) half of the patients (48%) responded to treatment, while the remaining patients experienced progression (52%). No patients discontinued treatment due to toxicity. In the second subgroup (n=10, PSA changed between -40 and +25%), disease progression was observed in 70% of patients, and treatment was discontinued due to toxicity in 30% of cases. In the subgroup where PSA increased by more than 25% (n=14), 71% of patients experienced progression, and 29% discontinued treatment due to toxicity. (**Figure 4**; **Table 3**). The difference in overall survival between ^177^Lu-PSMA RLT and ^225^Ac-PSMA RLT subgroups was statistically insignificant.

**Table 3:** Median overall survival in subgroups depending on PSA dynamics.

^225^Ac-PSMA RLT		^177^Lu-PSMA RLT		^225^Ac-PSMA RLT vs ^177^Lu-PSMA RLT	
*Median, 95%Cl*				*log-rank test*	*p-value*
PSA decline of ≥50%					
12.130		20.364		1.304	0.254
PSA changed between -40 and +25%					
5.227		6.850		1.258	0.262
PSA increased of ≥ 25%					
12.229		12.459		0.123	0.725
*log-rank test*	*p-value*	*log-rank test*	*p-value*		
10.87	.004	18.54	<. 001		

Hematological toxicity was observed in a significant number of patients treated with both ^225^Ac-PSMA and ^177^Lu-PSMA RLT. During the treatment in the ^225^Ac-PSMA group, 58% of patients experienced anemia of varying severity, while 42% of patients did not. Mild anemia was observed in 10 patients (23.8%), moderate anemia in 12 (28.6%), severe anemia in 2 (5%) patients, and 9 patients discontinued treatment due to progressive anemia (21%). Leukopenia was observed in 57% of patients, with 11 patients (26%) having grade 1 leukopenia, 12 (28.5%) patients having grade 2 leukopenia, and 2 (5%) patients having severe leukopenia. Decreased platelet count was detected in 20 (47.6%) patients. 11(26%) patients had 1st degree thrombocytopenia, 6 (14.3%) patients had 2nd degree thrombocytopenia, a total of 4 patients experienced severe thrombocytopenia, 3 (7%) patients had 3rd degree thrombocytopenia and 1 patient had 4th degree thrombocytopenia.

Patients treated with ^177^Lu-PSMA RLT showed a similar pattern of hematological toxicity (**Table 4**). 53 (66%) patients experienced anemia, of which 39 (44.3%) patients had mild anemia,11 (12.5%) patients had moderate anemia, and 3 (3.4%) patients had severe anemia. Leukopenia was recorded in 52 (59%) patients. Of these, 22 (25%) patients had grade 1 leukopenia. 12 (13.6%) patients had grade 2 leukopenia and 2 (2.3%) patients had grade 3 leukopenia. No patient had grade 4 leukopenia. Reduced platelet counts were present in 42 (47%) of treated patients. However, 36 (41%) experienced grade 1 thrombocytopenia and 5 (5.6%) experienced grade 2 thrombocytopenia. 1 (1%) patient experienced grade 4 thrombocytopenia. The results are summarized in **Table 3**.

**Table 4:** Hematological toxicity after ^225^Ac-PSMA and ^177^Lu-PSMA RLT.

Hematological adverse effects			^225^Ac-PSMA			^177^Lu-PSMA					z-test for total % of toxicity, p
Grade	1-2	3	4	5	Total	1-2	3	4	5	Total	
Anemia	22 (52%)	2 (5%)	0	0	24 (58%)	50 (57%)	3 (3.4%)	0	0	53 (66%)	0.204
Leukopenia	23 (54.5%)	2 (5%)	0	0	23 (57%)	34 (39%)	2 (2.3%)	0	0	52 (59%)	0.271
Thrombocytopenia	11 (26%)	3 (7%)	1 (2%)	0	20 (48%)	41 (47%)	0	1 (1%)	0	42 (47%)	0.194

No other cases of Grade 3 or higher adverse events were reported, nor were any serious adverse events reported. No treatment-related adverse events or deaths have been reported.

## Discussion

Some therapeutic agents are currently available for the treatment of mCRPC and have been proven to increase patient survival. ^225^Ac-PSMA RLT and ^177^Lu-PSMA RLT are mainly used in later stages of treatment, but some researches demonstrate the efficacy of these therapies in various patient populations, including early treatment and in patients with hormone-sensitive prostate cancer (9).

The treatment landscape for metastatic castration-resistant prostate cancer (mCRPC) includes systemic options such as taxane-based chemotherapy, abiraterone, enzalutamide, radium-223, and olaparib, which align with current clinical guidelines and have demonstrated survival benefits in various trials. However, these therapies can be associated with significant adverse effects or may prove insufficiently effective in certain patients (10, 11).

In this context, PSMA-targeted radioligand therapy has emerged as a promising precision oncology approach for mCRPC. This theranostic paradigm integrates diagnostic imaging and targeted treatment using the same PSMA-targeting ligand, first for tumor localization via PSMA positron emission tomography and subsequently for the delivery of cytotoxic radiation (8). The fundamental mechanism involves the specific binding and internalization of the radiopharmaceutical by PSMA-expressing cancer cells, leading to lethal DNA damage from localized ionizing radiation (12).

Recent advances in PSMA PET imaging, exemplified by the approval of tracers like ^68^Ga-PSMA-11 and ^18^F-DCFPyL, have enhanced the detection of metastatic disease (13, 14). PSMA PET/CT not only outperforms conventional imaging in sensitivity and specificity but also shows strong correlation with PSA levels, offering high prognostic value for treatment outcomes in mCRPC (15).

The two primary radionuclides for PRLT, lutetium-177 (^177^Lu) and actinium-225 (^225^Ac), offer distinct mechanisms. ^177^Lu emits medium-energy beta particles with a tissue penetration of up to 2 mm, creating a beneficial “crossfire” effect ideal for smaller lesions with homogeneous, high PSMA expression. In contrast, ^225^Ac decays via an alpha-particle cascade. These alpha particles have a very short path length (50–100 µm) but deliver high linear energy transfer (LET), causing dense, irreparable DNA double-strand breaks, making it particularly potent against larger, heterogeneous tumors or those with lower PSMA density (16, 17).

Clinical evidence supports the efficacy of both agents. Therapy with ^177^Lu-PSMA achieves a ≥50% PSA reduction in a substantial proportion of patients with mCRPC and has demonstrated a favorable toxicity profile (18, 19). The TheraP trial further established the superiority of ^177^Lu-PSMA over cabazitaxel in terms of PSA response, toxicity profile, and quality of life (20).

Emerging data suggest that ^225^Ac-PSMA may achieve higher biochemical response rates. A recent meta-analysis reported PSA declines of ≥50% in approximately 49% of patients treated with ^177^Lu-PSMA compared to 60% with ^225^Ac-PSMA (21).

Clinical studies and meta-analyses confirm that in mCRPC, [^225^Ac]Ac-PSMA therapy, based on alpha-particle emission, induces a more pronounced antitumor response compared to [^177^Lu]Lu-PSMA, which is objectively reflected in higher rates of PSA decline. The targeted action of alpha particles, which have an exceptionally short tissue range, is theoretically designed to minimize exposure to surrounding healthy tissues. Nevertheless, in clinical practice, characteristic adverse events such as grade 1/2 xerostomia and anemia are observed, associated with the non-selective irradiation of salivary glands and bone marrow (22, 23).

Furthermore, ^225^Ac-PSMA has shown clinical activity and acceptable toxicity in patients who have progressed on ^177^Lu-PSMA, indicating that these treatments are not mutually exclusive but can be sequenced (8). Importantly, while the shorter particle range of alpha-emitters might theoretically spare bone marrow, clinical observations indicate a different toxicity profile, with xerostomia being a prominent dose-limiting adverse event for ^225^Ac-PSMA (24).

Our study included patients with the advanced oncological process who had previously received other types of treatment, but the extension of RLT to patients with earlier stages of the disease, including *de novo* is becoming increasingly relevant, especially given the fact that patients who have previously received taxane-based chemotherapy have lower survival rates and treatment efficacy (25, 26).

Administering treatment at later stages of the disease—after progression—often results in a worsened somatic status, and the aggressive nature of mCRPC in these cases leads to poorer outcomes (12, 25). Therefore, therapies such as RLT, which are traditionally used as a last-line treatment, should be evaluated as an earlier-line therapy for patients exhibiting an initially aggressive course of prostate cancer (27).

Both ^225^Ac-PSMA RLT administered before or after ^177^Lu-PSMA RLT have shown promising results in several patients (20, 28). However, it is not fully understood which patient groups benefit most from treatment and what predictors of response to ^225^Ac-PSMA RLT after prior ^177^Lu-PSMA RLT exist.

Hematological toxicity is a well-documented side effect of 225AC-PSMA and ^177^Lu-PSMA RLT. The high incidence of this toxicity may be related to limited bone marrow reserve due to active disease progression at the time of therapy initiation, prior treatment, and comorbidities. Previous studies have shown that the level of toxicity affecting bone marrow during RLT is considered moderate, and therefore many advanced patients may be offered some form of RLT (28, 29). Since ^225^Ac-PSMA and ^177^Lu-PSMA RLT are typically administered as a last-line treatment for patients who are refractory or unsuitable for to other therapies, compromised functional bone marrow reserve and decreased renal function are frequently observed at the initiation of therapy (30, 31). Overall, our findings, consistent with data from other studies, indicate a moderate level of hematologic and renal toxicity even in patients with limited bone marrow and renal reserves at baseline (32, 33).

This study has important limitations, such as insufficiently balanced patient samples in the actinium and lutetium treatment groups and a limited number of patients in the ^225^Ac- PSMA RLT group. This study has several important limitations that should be considered. First, our analysis did not include a systematic assessment of some key treatment-related toxicities, specifically renal function, xerostomia, and fatigue. A comprehensive assessment of these side effects and their correlation with treatment cycles or cumulative administered activity was beyond the scope of this study. Second, the observational, non-randomized design of our study inherently limits the strength of our conclusions regarding the efficacy and safety of treatment. Also, an important limiting factor is that the study was conducted at a single research center. Given the importance of understanding the true tolerability profile and long-term safety, analysis of renal function and quality of life parameters (including xerostomia and fatigue) will be included in our future studies.

An important advantage of this study is the same source of radionuclide or peptide used to synthesize the therapeutic agent. All laboratory and instrumental examinations, including PET-CT, were performed at the same center, which reduces potential differences in performance.

In conclusion, actinium and lutetium-based treatment increases the life expectancy of prostatic cancer patients and should be administered in case of a high prevalence of the oncological process. However, further prospective studies comparing the effect of ^177^Lu- PSMA RLT and ^225^Ac- PSMA RLT are required to identify the groups of patients who benefit most from a particular therapy and to develop the most favorable sequence of their use. A more detailed assessment of the spectrum of adverse events that may be caused by the treatment is also an important area of study.

## Conclusion

This observational study suggests that ^225^Ac-PSMA and ^177^Lu-PSMA RLT offer comparable efficacy and safety for patients with mCRPC. The number of treatment cycles administered was a key factor associated with survival outcome. These results support the use of both agents in the therapeutic arsenal for mCRPC. Larger, randomized controlled trials are needed to definitively establish their comparative effectiveness, optimal sequencing, and position in the treatment landscape (12, 27).

## Data Availability

The raw data supporting the conclusions of this article will be made available by the authors, without undue reservation.
